# Structural mechanical properties of radiation-sterilized human Bone-Tendon-Bone grafts preserved by different methods

**DOI:** 10.1007/s10561-015-9538-1

**Published:** 2015-12-17

**Authors:** Grzegorz Gut, Joanna Marowska, Anna Jastrzebska, Ewa Olender, Artur Kamiński

**Affiliations:** Department of Transplantology and Central Tissue Bank, Medical University of Warsaw, Ul. Chalubinskiego 5, 02-004 Warsaw, Poland; National Centre for Tissue and Cell Banking, Chalubinskiego 5, 02-004 Warsaw, Poland

**Keywords:** Structural mechanical properties, Anterior cruciate ligament reconstruction, Bone-Tendon-Bone allografts, Glycerolization, Lyophilization, Radiation-sterilization

## Abstract

To avoid the risk of infectious disease transmission from donor to recipient, allografts should be terminally sterilized. In the previous paper (Kaminski et al. in Cell Tissue Bank 10:215–219, 2009) we presented the effect of various methods of preservation (deep fresh freezing, glycerolization, lyophilization), followed by irradiation with different doses of electron beam (EB), on material (intrinsic) mechanical properties of human patellar tendons cut out as for anterior cruciate ligament reconstruction, obtained in failure tensile test. As structural mechanical properties are equally important to predict the behaviour of the graft as a whole functional unit, the purpose of the present paper was to show the results for failure load and elongation, obtained in the same experiment. Paired Bone-Tendon-Bone grafts (BTB) were prepared from cadaveric human patella tendons with both patellar and tibial attachments. They were preserved by deep freezing, glycerolization or lyophilization and subsequently EB-irradiated with the doses of 25, 35, 50 or 100 kGy (fresh-frozen grafts) or a single dose of 35 kGy (glycerolized and lyophilized grafts). Each experimental (irradiated) group was provided with control (non-irradiated), donor-matched group. The specimens from all groups were subjected to mechanical failure tensile test with the use of Instron system in order to measure their structural properties (failure load and elongation). All lyophilized grafts were rehydrated before mechanical testing. In our study we did not observe significant deterioration of structural mechanical properties of BTB grafts processed by fresh-freezing and then terminal sterilized with growing doses of EB up to 100 kGy. In contrast, BTB grafts processed by glycerolization or lyophilization and irradiated with 35 kGy showed significant decrease of failure load. Obtained results suggest that deep-frozen irradiated grafts retain their initial mechanical properties to an extent which does not exclude their clinical application. However, biomechanical investigations constitute only the first step to evaluate the potential clinical usefulness of such allografts and further extensive in vivo studies are needed.

## Introduction

The anterior cruciate ligament (ACL) is essential for maintaining the stability of the knee, particularly in activities involving cutting (sudden change in direction), pivoting and kicking (Mohtadi et al. 2011). ACL acts by restraining anterior translation of the tibia relative to femur (Seitz et al. 1996) and preventing abnormal rotational motion and varus/valgus angulation at full knee extension.

Anterior cruciate ligament tears are especially common in athletes such as football, basketball and volleyball players, as well as Alpine skiers, with usually much higher incidence ratio observed in females compared to males (Prodromos et al. 2007).

In ACL reconstructive surgery both autografts and allografts are used, with no sufficient evidence of the advantage of the first approach over the second one in terms of clinical outcomes, if no terminal sterilization with gamma irradiation has been applied (McDermott and Thomas 2005; Hu et al. 2013). However, to avoid the potential of infectious disease transmission from a donor to a recipient (Eastlund 2006; Segur et al. 1998; Bohatyrewicz et al. 2006; Ireland and Spelman 2005; Schubert et al. 2012; Terzaghi et al. 2015; McDermott and Thomas 2005) and ensure their microbial safety (Sterility Assurance Level 10^−6^), allografts should be radiation sterilized with gamma radiation or accelerated electron beam (EB), usually with the doses of 25 or 35 kGy. In recently published retrospective analysis of bone and tendon allografts, obtained from 196 organ and tissue donors between 2008 and 2011, the overall incidence of bacteriological contamination was 23 % (Terzaghi et al. 2015), whereas in other studies it ranged from 5.8 % for musculoskeletal allografts (Ireland and Spelman 2005) to 48 % for bone allografts from cadaveric donors (Bohatyrewicz et al. 2006). These data clearly indicate the importance of terminal sterilization of tissue allografts procured from cadaveric donors.

Terminal sterilization of tissue allografts using ionizing radiation is supposed to adversely affect their initial biomechanical properties due to both direct (Kempner 2001, 2011) and indirect effects (Hammad 2008), resulting in damage of macromolecules important for graft structure and quality, like collagen and noncollagenous proteins (Bailey 1968).

In non-irradiated, intact ACL obtained from young adult cadaveric donors (mean age 26 ± 6 years), maximum axial load before failure in high-strain tensile test was found to constitute 1725 ± 269 N (Noyes et al. 1984). The authors compared mechanical properties of intact ACL itself with various ligament graft tissues, used for ACL reconstruction and harvested from similar young-adult donor population. They found 14–15 mm wide bone-patellar tendon-bone grafts, medial or central portion, to be the strongest tissue tested, providing, respectively, mean maximum load equivalent to 159 and 168 % of that of ACLs (Noyes et al. 1984). In another study, performed on grafts from young cadaveric donors (mean age 28 years), Cooper et al. (1993) obtained similar values of mean ultimate load for 10 mm wide central third of bone-patellar tendon-bone composite, constituting 2977 ± 516 N. However, Matava and Hutton (1995) reported much lower values of maximum force (mean 1411.7 ± 574.9 N) in tensile test for the central one-third patellar tendons (tissue width ranging from 8 to 11 mm), obtained from donors at the age range from 24 to 45 years.

Although biomechanical parameters are essential for preventing premature failure of Bone-Tendon-Bone (BTB) allografts, these grafts meet also other important criteria of successful reconstructive surgery, as they provide rigid bone-to-bone fixation, resulting in rapid bone-to-bone healing and revascularization (Stecker and Parker 1999). In consequence, BTB allografts are regarded as the “gold standard” for ACL reconstruction (Stecker and Parker 1999).

The growing interest in clinical application of BTB allografts for ACL reconstruction resulted in extensive human and animal studies during the last two decades and a number of publications, concerning the effect of different processing methods of allografts, including sterilization by gamma radiation or accelerated EB, on their initial biomechanical properties (Rasmussen et al. 1994; Salehpour et al. 1995; Fideler et al. 1995; Hoburg et al. 2011; Samsell and Moore 2012; Yanke et al. 2013; Hoburg et al. 2014). In the majority of these publications, both structural (extrinsic) and material (intrinsic) mechanical parameters (Einhorn 1992; Turner and Burr 1993) were discussed, as they represent different aspects of the allograft biomechanical competence. Mechanical behaviour, and, in consequence, the quality of the whole structure essential for successful grafting, reflects not only its dimensions and geometry, but also potential changes at the tissue level in various components (mainly proteins), resulting from processing and radiation sterilization. Therefore, studying of both structural and material mechanical parameters gives better insight into the mechanism of mechanical properties deterioration.

It should be kept in mind, however, that reliable comparing of allograft structural mechanical properties prepared by different methods is inevitably associated with strictly defined methodological demands, concerning the homogeneity of their shape, dimensions and origin (Einhorn 1992; Turner and Burr 1993). In our previous paper (Kaminski et al. 2009) we have only published the results of tensile strength (material property) of human BTB allografts preserved by deep freezing, glycerolization or lyophilization, and subsequently radiation sterilized with accelerated EB. As we applied pairs of donor-matched BTB allografts in all our experimental and control groups, in contrast to commonly used randomly selected specimens, and we carefully controlled graft dimensions to provide their homogeneity, it seems justified to present also the results of structural properties obtained in that study. By publishing these results we would like to join the discussion on the effect of different preservation and terminal sterilization methods on BTB grafts quality.

## Materials and methods

The study was carried out as described in details earlier (Kaminski et al. 2009). Briefly, matched paires of patellar tendons were harvested within 48 h after death from 25 male cadaveric donors aged 17–59 years (mean 37.1 ± 13.5 years). BTB allografts with both patellar and tibial attachments were prepared by removing lateral parts of both tendons and bone attachments, leaving approximately central one-third of the initial tendon width. The grafts were assigned to seven experimental and control groups as described in Table 1. To provide reliable detection of the possible effects of processing and terminal sterilization methods on BTB graft biomechanics in groups with relatively small number of specimens, the specimens in control and experimental groups were donor-matched, i.e. BTB graft obtained from the left knee of a given donor was always assigned to the control group, whereas obtained from the contralateral knee—to the experimental group. As structural mechanical properties of BTB allografts were studied, special attention was paid to homogeneity of patellar tendon dimensions (length, width, thickness, and cross-sectional area), which were carefully measured using caliper, with the accuracy of approximately 0.1 mm.

**Table 1:** Description of experimental groups

Preservation	Sterilization dose and conditions	*N*
Fresh-freezing	25 kGy, DI	6
Fresh-freezing	35 kGy, DI	4
Fresh-freezing	50 kGy, DI	5
Fresh-freezing	100 kGy, DI	2
Glycerolization (GLY)	35 kGy, DI	4
Lyophilization (L)	35 kGy, AT	4

The groups of specimens from the first part of the experiment, aimed to evaluate the effect of irradiation dose on mechanical properties of fresh-frozen BTB allografts, were preserved by deep freezing (−70 °C) and subsequently EB-irradiated (on dry ice) with the doses of 0 kGy (control specimens), 25, 35 or 100 kGy.

The groups of specimens affiliated to the second part of the experiment, aimed to examine the effect of irradiation on mechanical properties of BTB allografts preserved prior to irradiation by glycerolization or lyophilization, were both EB-irradiated with the dose of 0 kGy (control) or 35 kGy (experimental). In groups preserved by glycerolization EB irradiation was performed on dry ice, whereas in preserved by lyophilization—at room temperature. Glycerolization was performed by incubation of BTB grafts in 10 % glycerol for 1 h. Subsequently, glycerolized specimens were frozen and stored at −70 °C with glycerol solution, whereas lyophilized ones were stored at room temperature until irradiation. The residual water content in lyophilized grafts was less then 5 %.

Electron beam irradiation was performed using an EB accelerator (LAE-10; 10 MeV) at the Institute of Nuclear Chemistry and Technology (Warsaw, Poland).

All experimental and control BTB allografts were subjected to failure tensile test with the use of Instron testing machine (USA). Before mechanical testing, frozen specimens were thawed at room temperature, whereas lyophilized ones were rehydrated by overnight incubation in buffered saline to enable regaining their initial wet weight. During mechanical testing, BTB allografts were fixed on Instron testing machine by specially designed clamps, limited to patellar and tibial bone fragments and leaving the tendon free along the whole length (including bone attachments), eliminating slippage phenomenon while allowing simulation of mechanical challenges/loads prevailing in the natural environment of the recipient knee. All tensile tests were performed at the cross-head speed of 50 mm/min. Registered data included maximum load at failure, as well as elongation at failure, defined as the difference (in mm) between final and initial length of the tendon. Relative elongation was calculated as the percent change in final tendon length as compared to the initial length.

Significance of differences between corresponding donor-matched control and experimental groups was evaluated by Student’s *t* test. Statistical calculations were performed using Statistica software, version 10 (StatSoft Inc., USA). In all analyses, *p* value ≤0.05 was considered to be statistically significant.

## Results

**Fig. 1:**
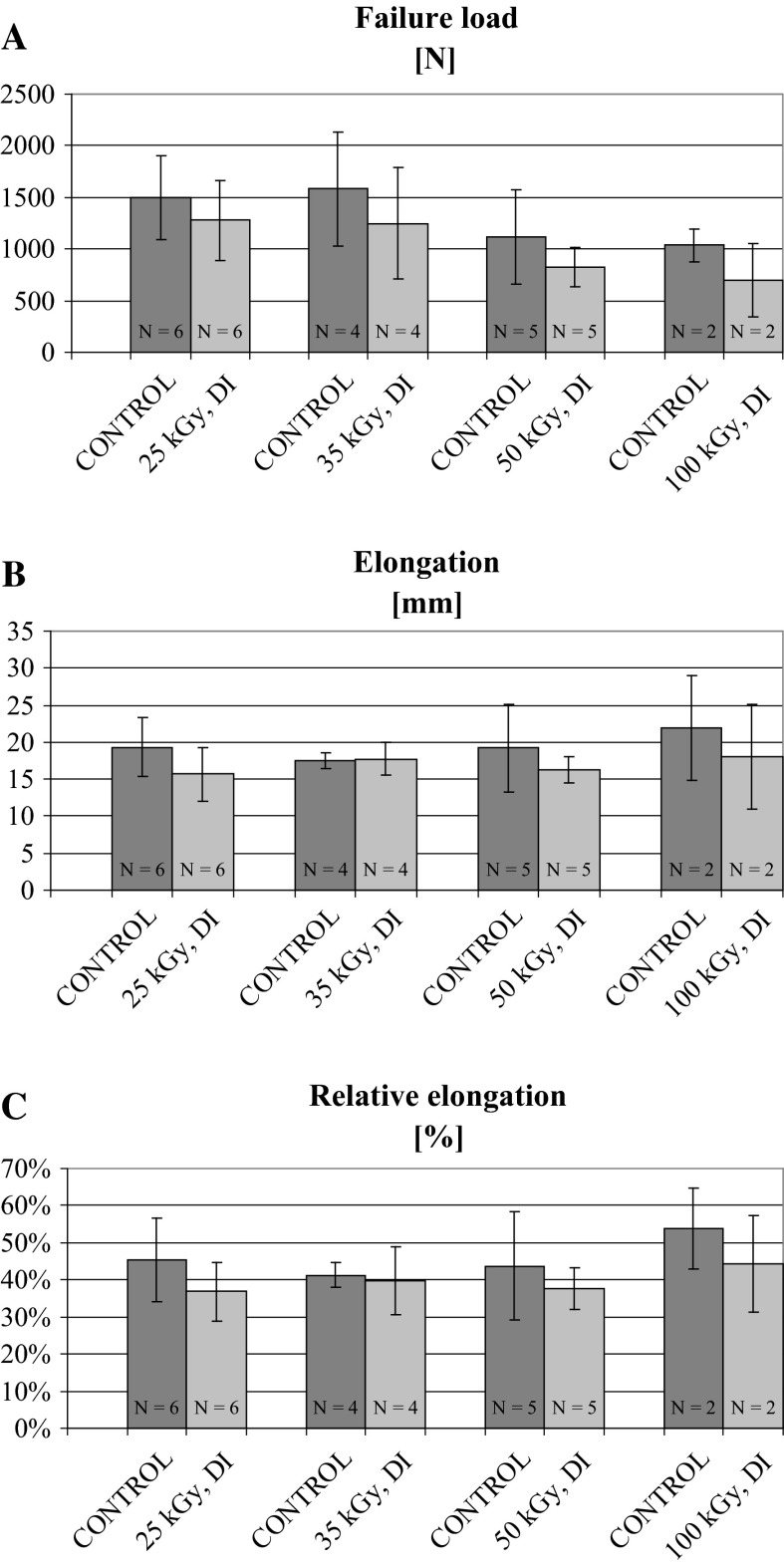
Failure load (**a**), elongation (**b**) and relative elongation (**c**) under tensile test of fresh-frozen donor-matched BTB control (non-irradiated) and EB-irradiated experimetal grafts. Doses applied were: 25, 35, 50 and 100 kGy. Data are shown as mean ± SD. No significant differences between control and irradiated groups were observed

**Fig. 2:**
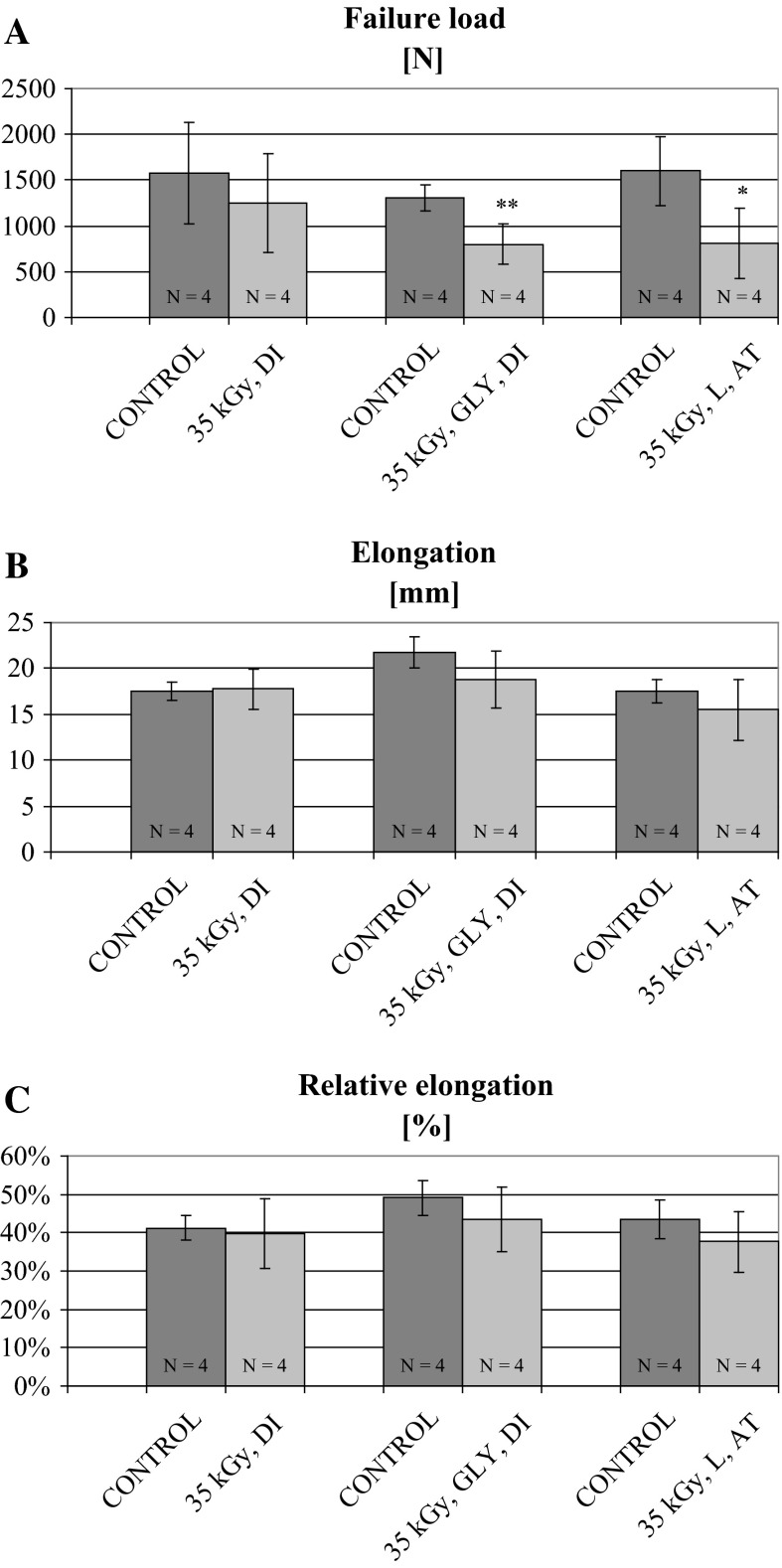
Failure load (**a**), elongation (**b**) and relative elongation (**c**) under tensile test of donor-matched BTB control (non-irradiated) and EB-irradiated (35 kGy) experimental grafts, according to different processing methods: fresh-freezing, glycerolization (GLY) or lyophilization (L) prior to irradiation. Data are shown as mean ± SD. Significant differences are marked with* asterisks*: *p = 0.026; **p = 0.008

The results are presented in Table 2 and in Figs. 1 and 2.

**Table 2:** Comparison of patellar tendon dimensions from donor-matched control (non-irradiated) and experimental (irradiated) BTB allografts before mechanical testing

Groups	*N*	Patellar tendon dimensions			
		Length (mm)	Width (mm)	Thickness (mm)	Cross-sectional area (mm^2^)
Control for 25 kGy	6	43.0 ± 4.4	11.5 ± 2.2	4.8 ± 0.8	55.2 ± 11.4
25 kGy	6	42.7 ± 4.5	11.2 ± 2.6	5.0 ± 0.6	54.8 ± 7.5
Significance		NS	NS	NS	NS
Control for 35 kGy	4	42.5 ± 1.9	11.0 ± 0.8	5.0 ± 0.0	55.0 ± 4.1
35 kGy	4	45.8 ± 6.3	10.5 ± 1.9	4.9 ± 1.0	50.5 ± 11.0
Significance		NS	NS	NS	NS
Control for 50 kGy	5	44.8 ± 8.5	9.4 ± 1. 7	5.0 ± 0.0	47.0 ± 8.4
50 kGy	5	43.8 ± 8.1	9.8 ± 1.5	4.6 ± 0. 9	45.0 ± 10.3
Significance		NS	NS	NS	NS
Control for 100 kGy	2	40.5 ± 4.9	10.0 ± 0.0	4.5 ± 0.7	45.0 ± 7.1
100 kGy	2	40.0 ± 4.2	10.0 ± 0.0	4.5 ± 0.7	45.0 ± 7.1
Significance		NS	NS	NS	NS
Control, glycerolized	4	44.8 ± 6.8	10.0 ± 0.0	4.8 ± 0.5	47.5 ± 5.0
35 kGy, glycerolized	4	43.8 ± 6.7	10.3 ± 1.7	5.3 ± 0.5	54.3 ± 13.4
Significance		NS	NS	NS	NS
Control, lyophilized	4	40.5 ± 4.7	11.8 ± 1.5	4.8 ± 0.5	56.0 ± 10.7
35 kGy, lyophylized	4	41.3 ± 4.3	10.0 ± 2.3	5.0 ± 1.4	49.0 ± 12.4
Significance		NS	NS	NS	NS

*NS* non-significant

The results of the comparison of patellar tendon dimensions between respective control (non-irradiated) and experimental (irradiated) groups are shown in Table 2. No significant differences were found in their length, width, thickness, and cross-sectional area. Therefore, the prerequisite justifying direct evaluation of BTB allograft structural mechanical properties was met in our experiment.

Special clamps for bone attachments applied in our study to fix BTB allografts on testing machine enabled the observation of the mechanism of specimen failure during tensile test. In the majority of tested specimens, BTB allograft failure resulted from simultaneous damage in bone attachment areas (bone fracture) and tendon fiber rupture. Failures limited to tendon only were incidental.

Results of the first part of the experiment, concerning the effect of EB irradiation dose on fresh-frozen BTB allograft failure load and elongation in tensile test, are shown in Fig. 1. EB irradiation resulted in slight, non-significant decrease in failure load in all experimental groups as compared to control ones (Fig. 1a). This decrease was most marked (however still non-significant) in the group irradiated with the highest dose of EB (100 kGy), where failure load constituted 68 % of the control value, whereas in the remaining groups was similar and dose-independent, ranging from 79 (35) to 85 % (25 kGy) of control. However, the results for the group irradiated at 100 kGy should be treated with caution due to the small number of specimens in experimental and control groups (Table 2), substantially limiting the field for interpretation.

No significant effect of EB irradiation on specimen elongation during failure tensile test was observed, although in three out of four studied groups it was slightly lower in irradiated specimens (Fig. 1b). The most marked decreases in elongation were obtained for groups irradiated with the lowest dose of 25 kGy (18.8 %) and with the highest dose of 100 kGy (18.2 %), as compared to their donor-matched control groups, indicating that changes in elongation were not dose-dependent. In absolute values, the elongation ranged from 17.5 to 22.0 mm in control groups, whereas in EB-irradiated ones from 15.7 to 18.0 mm.

Similarly, no significant differences in relative elongation (percent change in tendon length at failure as compared to its initial length) of BTB allografts were observed between control and experimental (irradiated) groups, although in irradiated groups this parameter showed slight tendency towards lower values (Fig. 1c). This tendency was especially marked in the group irradiated with 100 kGy, with the difference constituting nearly 10 %. In remaining pairs of control and experimental groups it ranged from approximately 1.6 (35) to 8.6 % (25 kGy) and seemed to be dose-independent. Relative elongation of BTB allografts ranged from 41.2 to 53.6 % in control groups and from 36.8 to 44.3 % in experimental, irradiated groups.

Results of the second part of the experiment, referring to the effect of different processing methods, namely glycerolization or lyophilization, applied before EB irradiation with a standard dose used in our Tissue Bank (35 kGy), on donor-matched BTB allograft failure load and elongation in tensile test, are shown in Fig. 2. Irradiation of glycerolized specimens were performed on dry ice (DI), whereas lyophilized ones at ambient temperature (AT). To enable direct comparison, results obtained for fresh-frozen control and experimental (irradiated with the same dose of 35 kGy) groups from the first part of the study, are shown in Fig. 2 as well. Both processing methods, i.e. glycerolization and lyophilization, applied for BTB allografts prior to irradiation, resulted in significant, marked decrease in failure load of irradiated specimens, while in fresh-frozen groups only non-significant, small decrease in this mechanical parameter was found (Fig. 2a). The observed decreases in failure load reached nearly 40 and 50 % of the control values in glycerolized and lyophilized experimental groups, respectively, whereas in fresh-frozen one only approximately 20 %. Irradiation of lyophilized specimens in ambient temperature might, additionally, contribute to this dramatic decrease. The observed significant, irradiation-induced decrease in failure load in glycerolyzed or lyophilized BTB allografts was not associated with significant changes in their elongation and relative elongation (Fig. 2b, c). However, tendency towards lower values of these mechanical parameters were found in irradiated groups as compared to control ones, which was not detected in the fresh-frozen groups. Elongation was decreased by 13.8 % in glycerolized, and by 11.4 % in lyophilized experimental (EB-irradiated) groups, as compared to their donor-matched controls (Fig. 2b), whereas for relative elongation the respective values were 5.6 and 5.9 %.

## Discussion

Our present study was designed to evaluate, in tensile test, structural mechanical properties of 10 mm human BTB allografts, prepared from cadaveric donors for ACL reconstruction. Special attention was paid to meet all methodological criteria for correct testing the whole structures, which are the prerequisite for reliable comparing between groups. First, donor-matched pairs of BTB allografts were prepared from left and right knee of a given donor and assigned to control and experimental groups, respectively, instead of randomly selected specimens. Second, the dimensions of patellar tendons were carefully measured during graft processing to provide their homogeneity, which resulted in the lack of significant differences between relevant control and experimental groups.

The objective of the first part of our study was to evaluate the effect of irradiation dose on failure load and elongation of BTB allografts preserved by fresh-freezing. The range of accelerated EB doses included 0 kGy (control specimens), and 25, 35, 50 and 100 kGy (experimental specimens). The second part of the study focused on the effect of one EB dose (35 kGy) on structural mechanical parameters of BTB allografts preserved with methods different from fresh-freezing, namely glycerolization and lyophilization. Moreover, special clamps for bone attachments used in our study to fix BTB allografts on the testing machine enabled direct observation of the process of graft failure during tensile loading. Due to the technique applied we showed that the mechanism of the majority of specimen failure included simultaneous damage in tendon-bone attachment areas (bone fracture) and intratendinous rupture, indicating the weakest parts of the graft. Failures limited to tendon only were incidental.

In our study, irradiation of fresh-frozen BTB allografts with growing doses of EB prior to mechanical tensile test resulted in the relatively slight decreases in failure load (Fig. 1) as compared to non-irradiated control ones, which constituted approximately 15 % at the lowest dose (25 kGy) and 32 % at the highest dose (100 kGy). These decreases were not statistically significant at any of the applied EB dose, and no consistent dose-dependent relationship was observed as failure loads of specimens irradiated with 35 and 50 kGy were diminished to the similar extent (21 and 18 %, respectively). However, the results obtained with the highest dose applied should be treated with caution due to small number of specimens in experimental and control groups. In contrast, both other processing methods studied, i.e. glycerolization and lyophilization of BTB allografts prior to irradiation with a single dose of 35 kGy, resulted in the much more marked and significant decreases of failure load, constituting approximately 40 and 50 % of the control values, respectively. It means that the reduction of this structural mechanical parameter was at least doubled as compared to the fresh-frozen specimens irradiated with the same dose. Irradiation of lyophilized BTB allografts in ambient temperature instead of temperature of dry ice could contribute additionally to the deterioration of their mechanical properties (Hamer et al. 1999).

The results of our study are somewhat controversial as the irradiation-induced decreases of fracture load observed by us were lower than reported by other investigators. Rasmussen et al. (1994), using pairs of frozen human patellar tendon-bone ligament allografts exposed to 0 or 4 Mrad (40 kGy) of gamma irradiation sterilization, registered 26 % reduction in maximum force during failure testing. In the goat model animal study, Gibbons et al. (1991) found significant decrease (27 %) in maximum force of paired frozen bone-patellar tendon-bone specimens exposed to 30 kGy gamma irradiation, whereas irradiation with 20 kGy did not alter significantly their mechanical properties. Also Salehpour et al. (1995), who loaded to failure in tension frozen pairs of goat patellar tendon-bone specimens bisected longitudinally into halves serving as control or irradiated with 40, 60 or 80 kGy, found significant, 46 % decrease in maximum force at the dose of 40 kGy, and dose-dependent reduction in mechanical properties of tested specimens. Similar reductions of initial maximum force were observed by Fideler et al. (1995) for fresh-frozen 10 mm bone-patellar tendon-bone allografts from young human donors, tested to tensile failure after gamma irradiation with 20, 30 and 40 kGy. These authors found significant (even for the dose of 20 kGy), dose-dependent decreases in ultimate load, which were found below reported values for the human ACL gamma-irradiated with 40 kGy (decrease up to 46 % of the initial value). However, Greaves et al. (2008) did not observe significant reduction in failure load of donor-matched pairs of human tibialis tendon allografts (single stranded and double stranded) following gamma irradiation in dry ice with the low dose (14.6–18.0 kGy). Moreover, no significant adverse effect of donor age up to 65 years was detected. Similar results were reported by Balsly et al. (2008), who investigated mechanical properties of donor-matched bone and soft tissue allografts, including BTB patellar tendon grafts, gamma irradiated on dry ice with two absorbed doses—low dose (18.3–21.8 kGy) and moderate dose (24.0–28.5 kGy). For BTB allografts the authors did not find statistical difference between control and low dose experimental groups both in structural (maximum load) and material (maximum tensile strength and modulus of elasticity) mechanical parameters studied. For the moderate dose significant reduction was observed only in tensile strength but not in maximum load and modulus of elasticity. However, these results seem to be somewhat confusing as mean cross-sectional area in the experimental group was much higher than in control, non-irradiated group, and could adversely affect the final statistical outcome (tensile strength was calculated by dividing the maximum load by the cross-sectional area of each specimen). In the remaining soft tissue allografts studied (anterior tibialis tendons, semitendinosus tendons and fascia lata) no significant differences in tensile strength were reported, irrespectively of gamma irradiation dose applied (Balsly et al. 2008), further justifying such interpretation.

All publications cited above referred to terminal sterilization of soft tissue allografts by gamma irradiation. However, since common acceptance of terminal sterilization of tissue grafts by a beam of accelerated electrons (EB) in 1970s, a new perspective of investigation concerning the effect of irradiation on tissue graft biomechanical properties, including soft tissue allografts, has emerged. Different characteristics of accelerated EB interaction with the matter as compared to gamma rays seem promising in this field. According to that, several recent publications referred to the effect of terminal sterilization with accelerated EB, as an alternative to gamma irradiation, on biomechanical properties of patellar tendon allografts used for ACL reconstruction. Hoburg et al. (2010) evaluated 10-mm wide human bone-patellar tendon-bone (BPTB) grafts and found no significant effect of EB irradiation in dry ice with the doses of 15 and 25 kGy on failure load and stiffness at time zero. However, irradiation with the high dose of 34 kGy resulted in significant decrease (approximately 20 %) in failure load as compared to non-irradiated, control grafts (1300.6 ± 229.2 vs. 1630.5 ± 331.1 N). In another study of the same group (Hoburg et al. 2014), in which the authors compared biomechanical properties of human BPTB grafts sterilized with EB or gamma irradiation at medium (25 kGy) or high doses (34 kGy), that decrease in failure load in relevant groups (EB 34 kGy vs. control) was slightly more marked (1139 ± 445 vs. 1741 ± 304 N, respectively) and statistically significant, whereas in the group irradiated with EB at medium dose (25 kGy) mean fracture load was lower as compared to control values, but the difference was not significant. As expected, EB irradiation was found to be less detrimental to biomechanical properties of BPTB grafts than gamma irradiation, which was expressed in all parameters studied (Hoburg et al. 2014). An order of magnitude of failure load as well as irradiation-induced decreases of this parameter value were similar to obtained in our study for fresh-frozen BTB grafts, both control and EB irradiated with the dose of 35 or 25 kGy (Fig. 1). However, in our study these decreases were not statistically significant, probably due to the higher dispersion of individual results around the mean value, reflected in relatively high standard deviation.

In efforts to reduce the detrimental effect of high-dose EB irradiation on structural biomechanical properties of fresh-frozen BPTB grafts, the same group performed a pioneer study, in which they evaluated fractionation of EB irradiation dose of 34 kGy, following the common procedure of radiotherapy in oncology, known to protect normal tissues against excessive damage, while not affecting the efficiency of malignant cell elimination (Hoburg et al. 2011). When the overall EB radiation dose of 34 kGy was delivered in ten portions of 3.4 kGy each, instead of a single dose, no significant differences were detected for any of the biomechanical parameters studied, including failure load, as compared to non-irradiated controls. In contrast, sterilization of BPTB allografts with both single-time EB and gamma irradiation resulted in significantly lower failure loads, but the observed decrease was relatively small for EB-irradiated specimens and much more pronounced when gamma irradiation was applied. However, as the authors mentioned, sufficient biomechanical properties of allografts constitute only one of several prerequisites of clinical success. Therefore, future studies concerning sterilization efficiency of multiple small EB dose, as well as the observation of postimplantation behaviour of grafts in vivo, are needed (Hoburg et al. 2011). That conclusion was supported by the elegant animal study of Schmidt et al. (2012), who investigated in vivo an early stage (6 and 12 weeks post surgery) of biological healing and restoration of the mechanical properties in female Merino mix sheep who underwent ACL replacement surgery with either a 34 kGy Ebeam treated free tendon allograft (single dose at the temperature of approximately −78 °C) or a non-sterilized fresh-frozen allograft, while native sheep ACL and hamstring tendons served as controls. The M. flexor digitalis superficialis, a model for hamstring tendons in humans, was used in the study, and outcomes included biomechanical testing (stiffness, ultimate failure load and AP-laxity), as well as histological analysis to investigate cell, vessel and myofibroblast density (Schmidt et al. 2012). Although the obtained results cannot be directly applied to Hoburg et al. (2011) study as irradiation was performed with a single Ebeam dose, they are relevant to other studies, including ours. The authors showed significantly altered Ebeam treated allograft remodeling with increased cellular repopulation and neovascularization, resulting in decreased biomechanical properties up to 12 weeks. In accordance with these findings, they did not recommend high dose Ebeam irradiation for soft tissue sterilization. However, the study of Schmidt et al. (2012) covered only the early stage of healing of free tendon allografts used for ACL reconstruction. In another in vivo animal study, performed on 60 skeletally mature foxhounds (Goertzen et al. 1995), deep-frozen bone-ACL-bone allografts were transplanted into right knees of dogs, thirty of them gamma-irradiated (under argon gas protection) with a dose of 2.5 Mrad (25 kGy). Examination of the allografts was conducted at 3, 6 and 12 months after implantation and included mechanical tensile testing, histology, collagen morphometry, neuroanatomical morphology and microvasculature. The authors did not find substantial differences in measured parameters between the experimental groups (non-irradiated and irradiated allografts) at any time-point of examination, except for slight hypervascularity in irradiated allografts as compared to non-irradiated ones at 12 months. However, at 12 months, no complete restoration of mechanical properties of both irradiated and non-irradiated allografts, as compared to normal (native) ACL, was observed (Goertzen et al. 1995). Keeping in mind all the differences between those in vivo studies, especially the irradiation source, irradiation dose applied under argon protection and observation period, it seems that the issue of soft grafts sterilization by high dose irradiation is still an open field for further investigations.

Analysis of elongation and relative elongation of fresh-frozen BTB grafts irradiated with growing doses of EB (25, 35, 50, 100 kGy) in the first part of our study revealed no significant differences in these structural parameters up to the highest dose applied, as compared to non-irradiated control groups. However, both elongation and relative elongation in groups irradiated with 25, 50 and 100 kGy showed tendency towards lower values than observed in donor-matched control groups. Although in recent works by Hoburg et al. (2010, 2014) results referring to this parameter itself were recorded, but not shown, we cannot compare them directly with our results. However, the authors did not find significant effect of EB irradiation with 34 kGy on stiffness, which is a derivative of failure load and elongation, defined as a slope of the linear part of the load/deformation curve (Einhorn 1992; Turner and Burr 1993). As they reported decreased failure load with that dose of EB, it implicated decreased elongation as well to maintain the slope unchanged. It seems, therefore, that the results of our study support the findings of Hoburg group (Hoburg et al. 2010, 2014).

In contrast to BTB grafts preserved by fresh-freezing prior to EB irradiation, those preserved by glycerolization or lyophylization in the second part of our study showed much more marked and significant decrease of failure load following irradiation with EB dose of 35 kGy. Elongation and relative elongation were also slightly, but not significantly diminished, which was not observed in fresh-frozenspecimens irradiated with the same dose. As values of failure load in control groups processed by glycerolization or lyophilization were situated well within the range obtained for fresh-frozen control group, the effect of these three procedures, if any, was comparable. However, both glycerolization and lyophilization, preceding EB irradiation of BTB allografts, seemed to enhance their radiosensitivity, and, in consequence, irradiation-induced deterioration of failure load. Irradiation of lyophilized specimens in ambient temperature might, additionally, contribute to this decrease.

When irradiation is performed in the relative absence of water due to lyophilization, macromolecules essential for graft biomechanical properties undergo irreversible breaking of covalent bonds and chain scissons, whereas crosslinking is much limited (Kempner 2001, 2011). This direct deteriorating effect of ionizing radiation is reflected by dramatic increase in collagen solubility in vitro as compared to grafts irradiated in aqueous solution (Dziedzic-Goclawska 2000; Dziedzic-Goclawska et al. 2005), when hydroxyl radicals, resulting from water radiolysis (indirect effect of ionizing radiation) are believed to enhance the crosslinking of collagen molecules (Hammad 2008; Kempner 2001).

While marked decrease in failure load of lyophilized BTB grafts following EB irradiation at ambient temperature could be expected, it was less clear for grafts incubated in 10 % glycerol prior to irradiation in a frozen state (on dry ice). Glycerol is known to have the stabilizing effect on enhanced temperature-induced and urea-induced denaturation of type I collagen, probably due to binding to the surface of the collagen molecule (Penkova et al. 1999). Human skin allografts cryopreserved in a solution of 20 % glycerol prior to gamma irradiation in dry ice did not show any histological, cytotoxicological or physical alterations compared to non-irradiated cryopreserved skin (Rooney et al. 2008). Bourroul et al. (2002) found no adverse effects of gamma and EB irradiation with the dose of 25 kGy on the characteristics of skin obtained from cadaveric donors and processed in 85 % glycerol, including it’s histological structure, anatomical integrity, and biomechanical properties in tensile test. Thus, the elucidation of negative glycerol effect on structural mechanical competence of BTB grafts, observed in our study, needs further experiments.

To conclude, the results of our in vitro study, although obtained for relatively low numbers of specimens in control and experimental groups, but performed on paired, donor matched specimens, suggest that human BTB allografts, preserved by deep-freezing and subsequently EB-irradiated with the dose of 35 kGy, retain their initial biomechanical properties to the extend sufficient for their clinical application. However, biomechanical investigations constitute only the first step to evaluate the potential clinical usefulness of such allografts and further extensive in vivo studies are needed.

## Abbreviations

ACL: Anterior cruciate ligament
BTB: Bone-Tendon-Bone graft
EB: Electron beam
Gy: Grey
AT: Ambient temperature
DI: Dry ice
